# Protective Effect of PBCA Nanoparticles Loaded with Thymulin Against the Relapsing-Remitting Form of Experimental Autoimmune Encephalomyelitis in Mice

**DOI:** 10.3390/ijms20215374

**Published:** 2019-10-29

**Authors:** Sergey M. Lunin, Maxim O. Khrenov, Olga V. Glushkova, Svetlana B. Parfenyuk, Tatyana V. Novoselova, Elena G. Novoselova

**Affiliations:** Institute of Cell Biophysics of the Russian Academy of Sciences, PSCBR RAS, Institutskaya str. 3, Pushchino, 142290 Moscow, Russia; xpehob2004@mail.ru (M.O.K.); glushckova@mail.ru (O.V.G.); lana_kras2@rambler.ru (S.B.P.); novossulova_t@rambler.ru (T.V.N.); elenanov_06@mail.ru (E.G.N.)

**Keywords:** EAE, thymulin, PBCA nanoparticles, signalling, IL-17A, interferon-γ, site-specific phosphorylation of a RelA/p65

## Abstract

Relapsing–remitting experimental autoimmune encephalomyelitis (rEAE) in mice is a model that closely resembles relapsing–remitting multiple sclerosis in humans. This study aims to investigate a new approach to modulation of the inflammatory response in rEAE mice using a thymic peptide thymulin bound to polybutylcyanoacrylate (PBCA) nanoparticles. PBCA nanoparticles were used to prolong the presence of thymulin in the blood. Cytokine levels in blood were measured by ELISA; NF-κB and SAPK/JNK cascade activation, as well as Hsp72 and p53 protein expression, were measured by Western blotting. Animal health statuses were estimated using severity scores. Results showed that the cytokine response in rEAE was multi-staged: an early phase was accompanied by an increase in plasma interferon-γ, while the interleukin (IL)-17 response was markedly increased at a later stage. The stages were attributed to rEAE induction and maintenance phases. Thymulin significantly alleviated symptoms of rEAE and lowered plasma cytokine levels both in early and later stages of rEAE, and decreased NF-κB and SAPK/JNK cascade activation. Thymulin modulated NF-kappaB pathway activity via site-specific phosphorylation of RelA/p65 protein (at Ser276 and Ser536). The effect of nanoparticle-bound thymulin was more pronounced than the effect of free thymulin. Therefore, PBCA–thymulin can be considered a prospective treatment for this pathology.

## 1. Introduction

Demyelinating autoimmune diseases are a group of diseases characterised by immune dysfunction that leads to activation of auto-aggressive clones of lymphocytes against myelin host components. These diseases include Devic’s disease, encephalitis periaxialis diffusa, and multiple sclerosis. Multiple sclerosis is one of the most widespread autoimmune diseases, affecting over one million people worldwide [1]. Furthermore, multiple sclerosis primarily afflicts young people. In multiple sclerosis, auto-aggressive T-cells attack their own myelin components in the brain and spinal cord, inducing inflammatory demyelination in the central nervous system that leads to such symptoms as paraesthesia, paraparesis, neuritis, or ataxia. Although multiple sclerosis is frequently characterised by periodic relapses and remissions, it gradually progresses over time, and at present there are no reported cases of full recovery from the disease. Today, immunotherapies are the most promising treatment for multiple sclerosis. Such therapies may include, for example, monoclonal antibodies. Monoclonal antibodies show efficacy in the treatment of the inflammatory phase of multiple sclerosis. However, the complications caused by antibodies are associated with significant immunological adverse reactions. These include development of neutralising immunogenicity, secondary immunodeficiency, and secondary autoimmunity. These complications can affect the risk–benefit balance of biological agents. In 2018, daclizumab, an anti-CD25 monoclonal antibody, was withdrawn from the market due to concerns about the development of severe, unpredictable autoimmunity [2]. Other approaches, such as corticosteroid administration, also have restrictions, related to side-effects or a lack of efficiency. Therefore, there is currently a need for new therapies that produce no adverse reactions but still effectively decrease the symptoms of demyelinating diseases.

One potential therapy that shows little or no adverse effects is the thymic peptide thymulin, a metallopeptide consisting of a nonapeptide (Glu-Ala-Lys-Ser-Gln-Gly-Gly-Ser-Asp) in complex with a zinc ion, which has been reported not only to regulate maturation of lymphocytes in the thymus, but also to modulate the immune and neuroendocrine systems’ activities [3]. Numerous data showed that thymulin exerts mostly inhibitory effects on the inflammatory immune response both in vitro and in vivo [4,5,6]. Thymulin also affects the neuroendocrine stress response system including its major pathway, the hypothalamic–pituitary–adrenal axis [3], and produces analgesic effects in inflammatory conditions [7]. Despite its systemic anti-inflammatory effects, thymulin is non-toxic at very large doses [8], and it has no marked effect on animal physiological status. We have previously studied the effects of thymulin in several models of chronic inflammatory conditions and obtained promising results. Specifically, thymulin produced a protective effect in endotoxemic mice [9]. Moreover, we have previously observed a significant anti-inflammatory effect of thymulin in severe and mild progressive forms of autoimmune encephalomyelitis [10,11]. The aforementioned features make thymulin, either alone or in combination with other therapies, a potential treatment for diseases characterised by immune imbalances. Mechanisms of the anti-inflammatory effect of thymulin so far are not known, however, we showed its effects on different signal proteins from the NF-kappaB pathway in septic and autoimmune inflammation [5,10,11]. Along with that, recently we have shown a regulation of the NF-kappaB pathway in experimental autoimmune encephalomyelitis (EAE) via site-specific phosphorylation of a RelA/p65 protein from the NF-kappaB family. Particularly, induction of EAE was accompanied by increased RelA/p65 phosphorylation at Ser276 in spleen cells, whereas maintenance of EAE was characterised by RelA/p65 phosphorylation at Ser536 [12]. A role of thymulin in the differential regulation of the NF-kappaB pathway in EAE may clarify its possible mode of action.

One disadvantage of thymulin and other thymic peptides is their very low half-life in plasma (less than 10 min) [13]. Additionally, endogenous thymulin concentrations in human blood decrease with age from about 2 pg/mL in neonates to about 1.5 pg/mL in children and adults up to the age of 20 years, and to about 0.4 pg/mL in adults between 21 and 65 years of age [14]. Thus, it may be desirable to restore thymulin levels during therapy or to increase the half-life of externally administrated thymulin to achieve its prolonged action in chronic diseases. Some researchers have tried to prolong the life of plasma peptides with peptide chain modifications. For example, Gao et al. have constructed a fusion peptide of two other thymic peptides, thymopentin and thymosin-α, with a longer residence time in plasma compared to both thymosin-α and thymopentin alone [15]. Another approach is related to gene therapy. A single injection of a recombinant adenoviral vector for thymulin into newborn nude mice with undetectable circulating levels of thymulin elicited long-term restoration of serum thymulin in these mutants. Furthermore, neonatal thymulin gene therapy in nude female mice has been found to prevent the ovarian dysgenesis that usually develops in 70-day-old female nude mice [16].

Yet another approach used to prolong the presence of the peptide and to allow its oral administration is the formation of nanoparticles containing thymus peptides. In vivo data showed that orally administered poly-butylcyanoacrylate (PBCA) nanoparticles containing the thymic hormone thymopentin had similar effects as intravenous thymopentin, but with enhanced bioavailability due to the nanoparticles. Thymopentin was released from colloidal PBCA-thymopentin nanoparticles within 48 h [17]. Recently, we adapted this method for thymulin and estimated the effects of intraperitoneally-administered PBCA–thymulin nanoparticles in mice with chronic septic inflammation, induced by gradually increasing doses of LPS. Our data demonstrated that thymulin, either free or PBCA-bound, alleviated fever, reduced apoptosis, increased splenic cell number, decreased cytokine production, and decreased Hsp72, Hsp90, and TLR4 expression, along with the activity of the several signalling pathways. We also demonstrated that PBCA-bound thymulin was more effective in several respects than free thymulin [9]. Therefore, our results revealed the great potential of nanoparticle-bound thymulin for treatment of chronic inflammatory conditions, including multiple sclerosis.

At the present time, several types of models of multiple sclerosis have been developed. The most commonly used model is an experimental autoimmune encephalomyelitis (EAE), which is induced by immunisation of mice with myelin-derived peptides together with potent adjuvants [18]. Depending on the induction protocol, the mice strain, and the immunogenic peptide used, several EAE forms are possible, with different characteristics. For instance, EAE induced in C57BL/6 mice shows a chronic progressive character, while in PL/J or B10.PL strains it demonstrates an acute progress, without any relapses. In the SJL mouse strain, the disease shows a course with intermittent relapses and remissions that are much like those in multiple sclerosis in humans [19]. It should be noted that effects of thymulin, free or bound to nanoparticles, were not studied using this autoimmune pathology. Therefore, the present study was aimed to investigate the effects of PBCA-bound thymulin on the relapsing–remitting form of EAE (rEAE) in mice, which closely resembles relapsing-remitting multiple sclerosis in humans.

## 2. Results 

### 2.1. Nanoparticles

**Figure 1:**
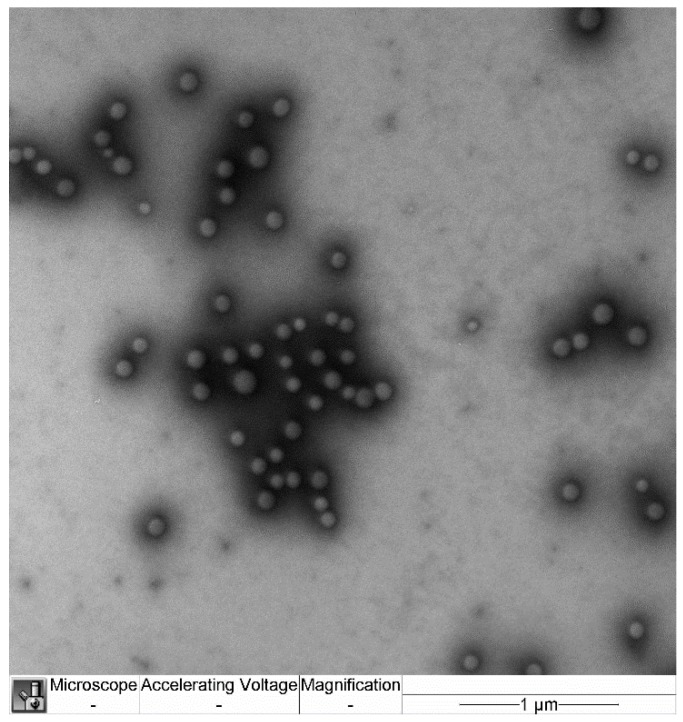
TEM micrograph of polybutylcyanoacrylate (PBCA)-thymulin nanoparticles. The scale bar represents 1 μm.

Sizes and morphology of nanoparticles are demonstrated in Figure 1. The average size of the nanoparticles was 156 ± 50 nm.

### 2.2. Relapsing-Remitting EAE

**Figure 2:**
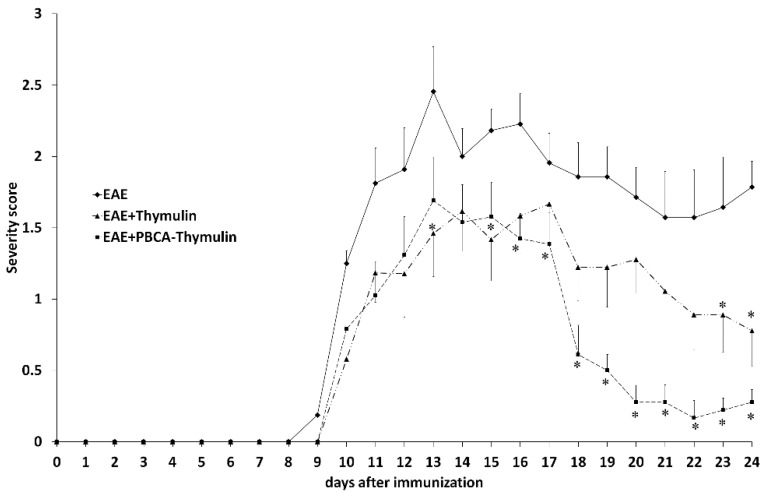
The severity scores of thymulin-treated, PBCA–thymulin-treated, and untreated mice with experimental autoimmune encephalomyelitis (EAE). Data from untreated mice (solid line) and mice treated with thymulin-bound polybutyl cyanoacrylate nanoparticles (PBCA–thymulin) or free thymulin (thymulin) every other day throughout the observation period, starting on the day 0 (the day of immunisation with proteolipid protein peptide). Data are presented as the mean severity score ± standard error of the mean for 10–20 mice per group, depending on the time period. Clinical signs of EAE were recorded every day. * Significantly different from untreated mice, according Mann–Whitney *U*-test, *p* < 0.05.

The proportion of mice excluded from the study (because they did not develop EAE) was rather low, only 5 of 60 (8.3%). We observed first symptoms of the pathology on the ninth day after the induction of the disease (Figure 2). The symptoms gradually increased for next five days (up to the 13th day), when the maximum severity score was observed. After this peak, the mice generally demonstrated some alleviation of symptoms. Subsequently, relapses and remissions were repeated several times in many animals. It should be noted that lethality in rEAE was low, and virtually all mice were still alive at the end of the observation period, although many had severe symptoms. The mice were sacrificed at different time points (the 12th, 18th, and 25th days of experiment) in subgroups consisted of five animals. To this end, the animals were selected that had the severity score closest to the mean score for the corresponding group at the corresponding time point.

The repeated intraperitoneal injection of free thymulin or PBCA–thymulin nanoparticles every other day significantly reduced the severity of the disease compared to untreated rEAE mice. In many cases a complete restoration was observed, while in the untreated rEAE group there were no full restoration cases (Figure 2). The time to peak score value was similar for untreated and treated mice with EAE. The effect of PBC-thymulin was more pronounced than that of free thymulin in the second half of the observation period

### 2.3. Cytokine Response

**Figure 3:**
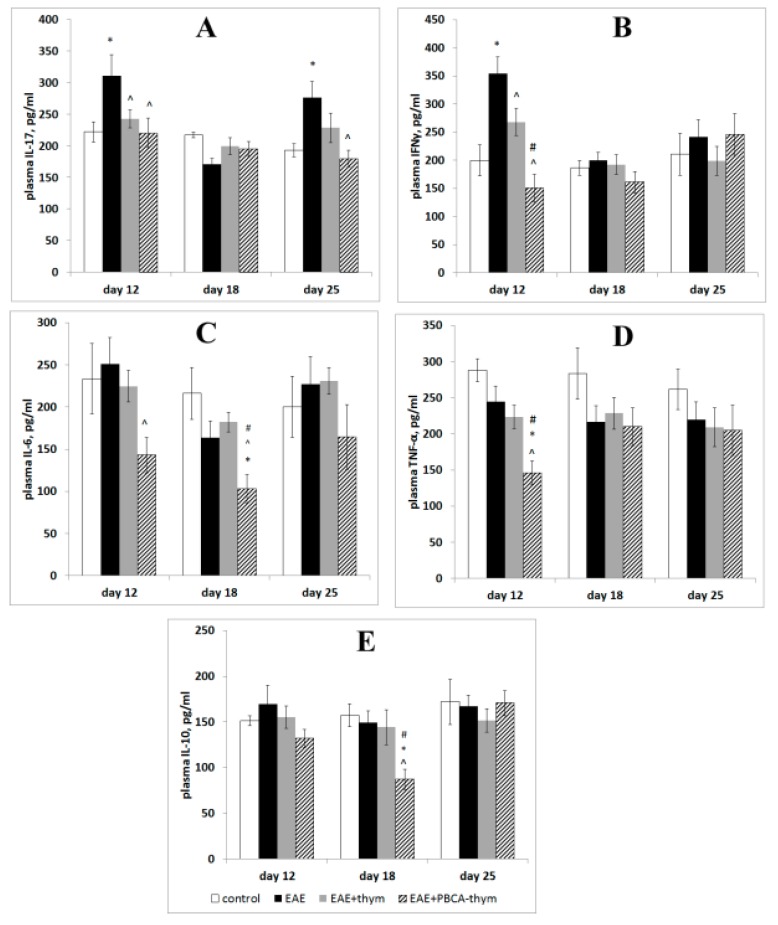
Plasma cytokine concentrations throughout the observation period. Animal groups: untreated experimental autoimmune encephalomyelitis (rEAE) mice, rEAE mice treated with PBCA–thymulin, and age-matched controls. (**A**) IL-17A. (**B**) Interferon-γ. (**C**) IL-6. (**D**) TNF-α. (**E**) IL-10. Measurements were made using ELISA at different times after immunisation (day 0). The values are expressed as an average of the means from five independent experiments (individual animals) (ng/mL) ± standard error of the mean. For each separate measurement, the samples were concurrently measured in quadruplicate. The averaged values from five experiments were analysed to determine the significance of the differences between groups (*n* = 5). Vehicle-treated age-matched animals served as controls. * Significantly different from the control group, *p* < 0.05; ^ significantly different from the EAE group, *p* < 0.05; # significantly different from the thymulin group, *p* < 0.05, according the Student’s *t*-tests, *p* < 0.05.

In order to investigate time features of the cytokine response, serum concentrations of the following cytokines were measured by the ELISA method at different stages of rEAE progression (days 12, 18, and 25): interferon-gamma (IFN-γ), interleukins 17A, 6, and 10 (IL-17A, IL-6, and IL-10), and tumour necrosis factor alpha (TNF-α). These cytokines are known to be produced by Th1 and Th17 subpopulations of T-helpers. The results demonstrated that in the early stages of disease (12th day after rEAE induction), concentrations of IFN-γ and IL-17A in the EAE group were increased by 40%–70% compared to healthy controls (Figure 3).

However, plasma IL-6, IL-10, and TNF-α were unaffected. Subsequently, plasma IL-17A and IFN-γ were normalised; moreover, IL-17A levels became lower than the control level. However, IL-17A increased again on the 25th day, whereas the concentrations of other cytokines were equal to those in the healthy mice. Thus, a biphasic cytokine response was observed, with an early phase characterised by an increase in plasma IL-17A and IFN-γ, and a later phase characterised by an increase only in plasma IL-17A. Therefore, our data using the rEAE model closely resembles the biphasic cytokine response recently observed in several acute EAE models, where an initial increase in IFN-γ was followed by an increase in IL-17A [10,20]. PBCA–thymulin nanoparticle administration exerted profound anti-inflammatory effects on IL-17A and IFN-γ levels, both in the early and later stages of rEAE. Moreover, the concentrations of plasma IL-6, TNF-α, and IL-10 decreased below control levels. It should be noted that free thymulin exerted a less profound effect than PBCA–thymulin.

### 2.4. Signalling Pathway Activity

**Figure 4:**
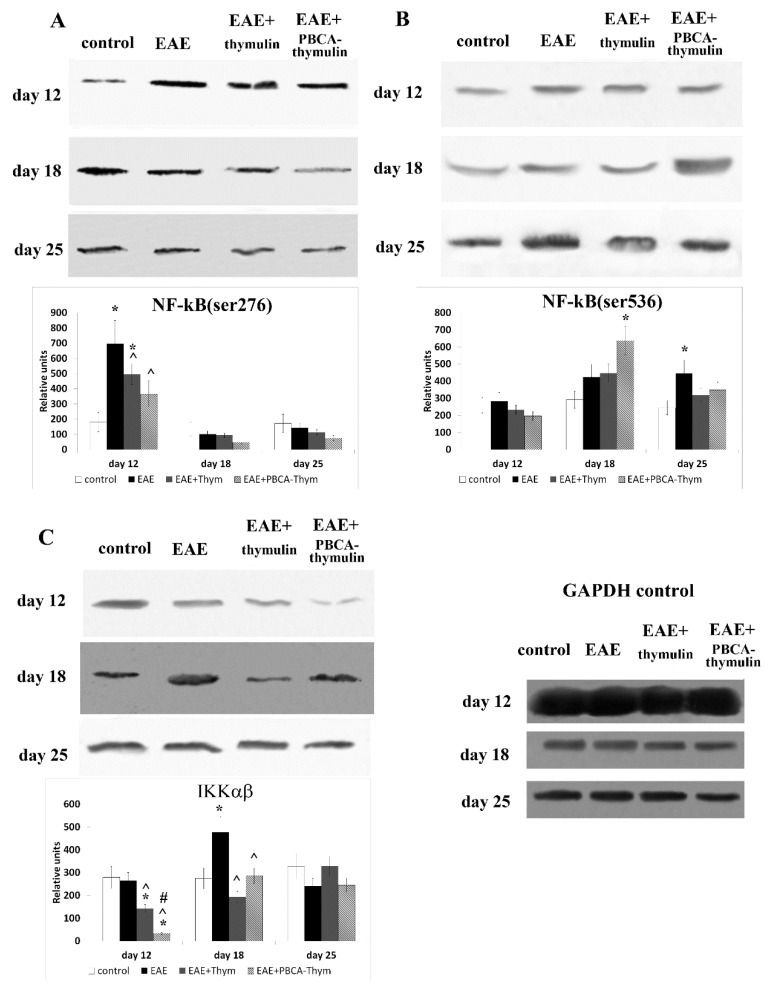
Effects of PBCA–thymulin on the activities of NF-κB signalling proteins: phosphorylation of p65/RelA at serine 276 (**A**) and serine 536 (**B**), and phosphorylation of IκB kinase (IKK) (**C**) in splenic lymphocytes throughout the course of disease. Animal groups: untreated mice (experimental autoimmune encephalomyelitis, rEAE), rEAE mice treated with PBCA–thymulin (rEAE + PBCA–thymulin), and age-matched controls. Protein levels were measured in spleen lymphocytes. Equal amounts of protein (10 μg) were analysed at different times after immunisation (day 0) by Western blot analysis using the corresponding antibodies. Histograms show the amounts of protein (± standard error of the mean) relative to those of the non-phosphorylated p65/RelA or IKK and internal GAPDH control (not shown), determined by protein blot densitometry using QAPA software from three independent experiments. * Significantly different from the control group, *p* < 0.05. ^ Significantly different from the EAE group, # significantly different from the thymulin group, *p* < 0.05, according the Student’s *t*-tests, *p* < 0.05.

The NK-κB signalling cascade plays a critical role in immune cell activation and inflammatory responses. The activation of this pathway can follow different routes involving different kinases. In particular, IκB kinase (IKK) may activate NK-κB through phosphorylation of the RelA/p65 protein at serine 536, while protein kinase A (PKA) may activate it through the phosphorylation of the RelA/p65 protein at serine 276. To investigate the effect of thymulin and PBCA–thymulin on the activity of these cascades in the cells from rEAE mice, we used spleen cells from the healthy control mice, the untreated mice with rEAE, and the mice treated with thymulin or PBCA–thymulin (Figure 4). Our results showed that in mice with rEAE, phosphorylation of the RelA/p65 protein at serine 276 was markedly (four-fold) increased compared to the healthy animals during the early stage of the pathology (12th day). At the phases, the phosphorylation of the RelA/p65 at serine 276 returned to control levels (Figure 4A). Treatment with thymulin or PBCA–thymulin significantly decreased phosphorylation at serine 276, and the decrease was by about two-fold.

Furthermore, we investigated the effects of these two forms of thymulin on phosphorylation of the RelA/p65 protein at another site, serine 536, which is supposed to be a target of protein kinase IKK. The results showed no changes in phosphorylation at serine 536 at the early phase of the disease (12th day), but gradual increase in levels of phosphorylation at serine 536 were observed at the later stages (18th and 25th days) (Figure 4B). The maximum increase was approximately 200% on the 25th day. Both thymulin and PBCA–thymulin had some effect on Ser536 phosphorylation. We observed a tendency towards a decrease in Ser536 phosphorylation on the later stage (25th day). Activation (phosphorylation) of the IKK, which is known to mediate the Ser536 phosphorylation, was not observed at the early stage of the disease (12th day), but was demonstrated at a later stage (18th day). However, at the latest stage (25th day), IKK phosphorylation decreased to normal levels (Figure 4C). Both thymulin and PBCA–thymulin led to a significant decrease in IKK activation in the cells from rEAE mice.

**Figure 5:**
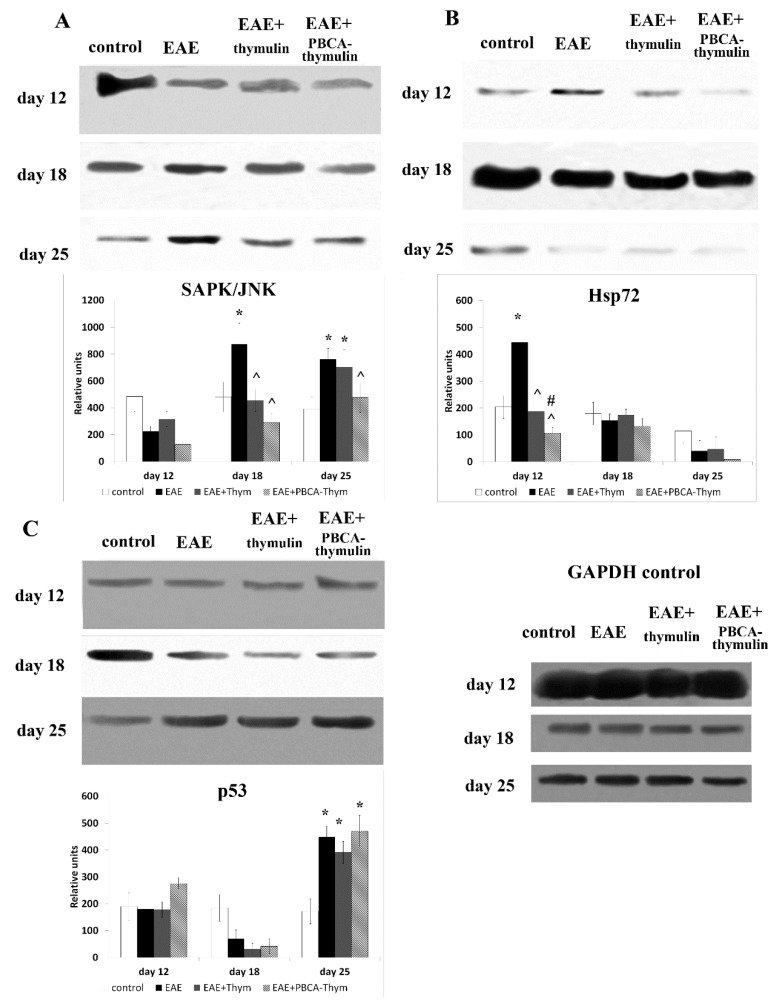
Effects of PBCA–thymulin on stress-related intracellular systems in cells from rEAE mice. Phosphorylation of SAPK/JNK (**A**) and p53 (**C**), and expression of Hsp72 (**B**) in splenic lymphocytes of treated and untreated mice throughout the course of disease. Animal groups: untreated mice (rEAE), rEAE mice treated with PBCA–thymulin (rEAE + PBCA–thymulin), and age-matched controls. Protein levels were measured in spleen lymphocytes. Equal amounts (10 μg) of protein were analysed at different times after immunisation (day 0) by Western blot analysis using corresponding antibodies. Histograms show the amount of protein (± SEM) relative to that of total SAPK/JNK, or p53, and internal GAPDH control (not shown), and are the results of protein blot densitometry measurements by QAPA software from three independent experiments. * Significantly different from the control group, *p* < 0.05. ^ Significantly different from the EAE group, *p* < 0.05, # significantly different from the thymulin group, *p* < 0.05, according the Student’s *t*-tests.

The stress-related SAPK/JNK pathway was markedly activated in untreated mice at the later stages of rEAE (Figure 5A). The increase was by about two-fold. PBCA–thymulin produced a significant inhibitory effect, decreasing SAPK/JNK phosphorylation to control levels, whereas the inhibitory effect of free thymulin was observed on day 18, but not on day 25.

The expression of an inducible form of heat shock protein 70 (Hsp72) also reflects the cellular stress condition. In contrast to SAPK/JNK activation, the expression of Hsp72 was increased at the earliest stage only, decreasing to control levels at later stages. Both thymulin and PBCA–thymulin completely blocked the rEAE-induced increase in Hsp72 expression (Figure 5C), which corresponds to our previous data showing that thymulin blocks inflammatory Hsp72 induction [4].

We also estimated the levels of phosphorylated and total p53 protein, using Western blotting method (Figure 5B). The increased phosphorylated-p53/total-p53 ratio in spleen lymphocytes suggested that this pro-apoptotic and cell cycle-regulating protein was only activated in the latest phase of rEAE (25th day), but not in the earlier phases, somewhat corresponding to SAPK/JNK activation. However, the administration of thymulin, both in free or nanoparticle-bound form, exerted no effects on this pro-apoptotic pathway.

Therefore, thymulin and PBCA–thymulin affected both early and delayed stress-related events in cellular signalling, and these changes led to a significant decrease in serum cytokines associated with autoimmune responses, and ultimately, to alleviation of disease symptoms. PBCA-bound thymulin was more effective in chronic inflammatory conditions than free thymulin. Therefore, protection of thymulin with PBCA-nanoparticles prolonged its effects associated to the immune response and alleviation of the symptoms.

## 3. Discussion

Multiple sclerosis is considered a classical T cell-mediated autoimmune disease with a complex genetic background. The mechanisms involved in producing the acute lesions of multiple sclerosis seem to share similarities with those described for acute EAE. It is now generally accepted, however, that in addition to the acute inflammatory component, a more insidious degenerative process also exists and contributes substantially to the progression of disability [1]. Therefore, multiple sclerosis can be considered a multi-staged process, with stages such as induction and maintenance. It is now generally accepted that Th1 and Th17 cells play a central role in disease development [21,22,23]. Th17 cells attach to brain endothelial cells at a higher rate than Th1 cells, in part due to the presence of CD146 on the surface of Th17 cells [24]. Moreover, Th17 cells express high levels of CCR6 and CD6, which enhance the entry of T-cells into the CNS [25]. Along with IL-17, Th17 cells produce interleukins 6, 9, 21, 22, 23, and 26, along with TNF-α, whereas the cytokine profile of Th1 cells includes IFN-γ, IL-10, and IL-2 [26]. Recently, using several acute, fast-progressing EAE models without intermittent relapses/remissions, we observed a multi-stage cytokine response, with early Th1 cytokine production (mainly IFNγ) and later Th17 cytokine production (mainly IL-17A). These stages may be either distinct or overlapping to some degree, depending on the rate of acute EAE progression [10,11].

The present data on long-lasting chronic rEAE characterised by periodic relapses and remissions also showed two peaks of cytokine response. We suppose that this type of autoimmune response may reflect a peculiarity of the models used, which may or may not be shared in the development of multiple sclerosis in humans. Specifically, rEAE is commonly induced in susceptible mice strains by administration of myelin components simultaneously with potent adjuvants (CFA with a high dose of *Mycobacterium tuberculosis* plus pertussis toxin). We suggest that the early, Th1-mediated stage of the cytokine response may reflect an immune response to these rEAE-inducing substances. Recently, using inhibitory analysis, we showed that this early autoimmune response was independent of NF-κB cascade activation through kinase IκB kinase (IKKαβ) [12]; however, it was characterised by phosphorylation of the p65/RelA protein of this cascade at Ser276, which is commonly attributed to protein kinase A (PKA). This is completely consistent with results from the present study on the Ser276 phosphorylation of p65/ReA. PKA is known to be involved in regulation of glucocorticoid receptor (GR) pathways [27]. The repression of GR activity by p65 requires PKA phosphorylation of Ser276, whereas GR-mediated inhibition of NF-κB activity is also PKA-dependent. Thus, the cross-repression of NF-κB and GR activity is regulated by PKA-associated signalling. It is possible that the initial stage of the EAE process is mediated through Th1 cells and reflects a break in immunological tolerance and escape from surveillance of the glucocorticoid system during EAE induction. Again, the induction stage of multiple sclerosis in humans may or may not be related to the above mechanisms. On the contrary, the maintenance stage of the autoimmune response in EAE may be mediated through Th17 cells that appear later, possibly as a result of Th1 cell activation. On an intracellular level, the later stages were characterised by NF-κB cascade activation through phosphorylation of p65 at Ser536, which may be attributed to IKK. This stage may reflect the “canonical” progression of multiple sclerosis, leading to symptoms associated with demyelination.

We have earlier shown that thymulin exerted anti-inflammatory effects in acute septic inflammations and the effects were associated to inhibition of IKKαβ activity and phosphorylation of p65 at Ser 536 [9]. The similar effect of thymulin on the IKKαβ was demonstrated in a progressive form of EAE [11]. It was shown earlier that binding to PBCA prolongs the life of thymic peptides in plasma from several minutes for the free peptide to 48 h for the bound peptide [17]. Therefore, we hypothesised that PBCA–thymulin would be effective for the treatment of prolonged chronic autoimmune inflammation, expressed as relapsing/remitting EAE.

In the present work we observed normalizing effects of free thymulin and PBCA–thymulin on phosphorylation of p65 at Ser 536. However, PBCA–thymulin exerted a significantly more profound effect on phosphorylation of p65 at Ser 276, which we attribute to the induction phase of EAE. Furthermore, PBCA–thymulin was more effective in alleviating an overall health condition of animals. Indeed, PBCA–thymulin nanoparticles exerted profound anti-inflammatory effects, decreasing the symptoms of the disease. In the PBCA–thymulin-treated group, symptoms decreased more rapidly and the magnitude of the effect was more profound than in thymulin-treated group. This effect was also reflected in both IFN-γ and IL-17A levels in the early and in the later stages of rEAE. Moreover, the concentrations of plasma IL-6, TNF-α, and IL-10 decreased below control levels. Therefore, nanoparticles affected both the Th1-mediated induction stage of rEAE and the Th17-mediated maintenance stage, as was confirmed by the decrease in the activation of the pro-inflammatory NF-κB signalling pathway in the splenocytes of nanoparticle-treated rEAE mice. It should be noted that PBCA–thymulin significantly decreased Ser276 phosphorylation in p65/RelA protein, which is attributed to PKA and associated with GR system repression. However, rEAE still developed in treated mice, though their peak on the plot of severity scores was lower than in untreated rEAE mice. At the later stage (the maintenance stage) the effect of PBCA–thymulin was more profound, and there were even cases of complete recovery (at least during the observation period). This was accompanied by decreased IKKαβ phosphorylation and IL-17 production. It is important to note that PBCA–thymulin nanoparticles had no effect on or reduced production of IL-10, which is known as an anti-inflammatory cytokine produced by Tr-1 cells, Tregs, CD39+ T cells, and also by B cells with regulatory phenotype. This finding might suggest that the protection observed is independent of Il-10-producing cells.

Moreover, the stress-related signal cascade SAPK/JNK, which was profoundly activated in the later stages of rEAE, was significantly suppressed by PBCA–thymulin treatment, but not free thymulin treatment. Stress-activated protein kinase c-Jun NH2-terminal kinase (SAPK/JNK) is activated by many types of cellular stress and extracellular signals, including UV and y-irradiation, protein synthesis inhibitors (anisomycin), hyperosmolarity, toxins, ischemia/reperfusion injury in heart attacks, heat shock, anticancer drugs (cisplatinum, adriamycin, or etoposide), ceramide, T-cell receptor stimulation, peroxide, and inflammatory cytokines, including TNFα [28]. The increased activation of SAPK/JNK in later stages of rEAE may reflect the negative effects of stress factors during prolonged inflammation. Obviously, the decrease of SAPK/JNK activation in nanoparticle-treated mice may be considered a positive effect during the pathology development.

In addition, we studied the effects of free and PBCA-bound thymulin on another stress-related protein that is part of the cellular defence system, heat-shock protein Hsp70. Hsp70 and its inducible form, Hsp72, act as molecular chaperones in protein folding, transport, and degradation. Hsp72 levels markedly increased during the induction stage of rEAE, but not during later maintenance stages. The observed rise in the level of Hsp72 may be mediated by PKA, since PKA is known to phosphorylate heat shock factor 1, which migrates into the nucleus and activates Hsp70 protein expression [29]. This is in agreement with the aforementioned increase in Ser276 phosphorylation of p65/RelA during the suggested “break of immune tolerance” stage, which is also thought to be mediated by PKA. Both thymulin and PBCA–thymulin significantly decreased Hsp72 expression in the rEAE mice. This effect correlated with the ultimately less-severe disease state, and may be explained by a weakened primary response to adjuvants. These results are consistent with a very recent study that showed that thymulin reduced thermal hyperalgesia and paw edema induced by CFA, as well as CFA-induced activation of microglia cells, phosphorylation of p38 MAPK, and the production of spinal pro-inflammatory cytokines (TNF-α, IL-6) [6].

Furthermore, we studied the effect of thymulin and PBCA–thymulin on the phosphorylation of p53 protein, which is a member of the pro-apoptotic cascade. Activation of the SAPK/JNK pathway may lead to the phosphorylation of p53, a pro-apoptotic protein known to be a SAPK/JNK pathway substrate [30]. Indeed, we observed p53 activation in mice with rEAE, but only at the latest stage of the observation period. Though decreased SAPK/JNK activation, thymulin and PBCA–thymulin did not affect p53 activation in rEAE. However, in the context of autoaggressive lymphocyte clonal expansion, maintenance of splenocyte apoptosis should be considered a desirable effect.

## 4. Materials and Methods

### 4.1. Animals

Female 3-month-old SJL/J mice (purchased from the Breeding Facility for Laboratory Animals, Pushchino, Russia) weighing 20–25 g were kept in standard laboratory conditions (20–22 °C, 10 h:14 h light:dark cycle, 65% humidity) and supplied with food and water ad libitum. Standard mouse food pellets contained a balanced diet with proteins, vitamins, and minerals. Experimental procedures were approved by the Institutional Ethical Committee (approval #57, 12/30/2011), and the experiments were performed in accordance with the Guidelines for Ethical Conduct in the Care and Use of Animals.

### 4.2. Thymulin or PBCA–Thymulin Preparation

The thymulin solution was prepared from a serum thymic factor (Abcam, Cambridge, MA, USA), to which an equimolar concentration of ZnCl_2_ was added. An optimised nanoprecipitation method was used to prepare the PBCA nanoparticles [17]. Briefly, thymulin (1.0 mg) was dissolved in 10 mL 2.5% Pluronic F-68 solution, and the pH of the solution was adjusted to 2.5 with 0.1 mol/L HCl. Next, 50 µL of BCA monomer (B. Braun, Melsungen, Spain) was slowly injected dropwise into the water phase while stirring. After stirring for 30 min, the pH value of the solution was adjusted to 7.8 with 0.1 mol/L NaOH, and stirring was maintained for another hour. The colloid was freeze-dried and stored at −20 °C before use or was used immediately. The entrapment efficiency of this process has been shown to be 90% [16].

### 4.3. Induction of EAE and Application of Free Thymulin or Nanoparticles Loaded with Thymulin

To induce acute EAE, 150 μg of proteolipid protein (PLP) peptide 139–151 (GenScript, Piscataway, NJ, USA) was emulsified with complete Freund’s adjuvant containing 4 mg/mL H37Ra (*Mycobacterium tuberculosis*; Chondrex, Redmond, WA, USA). As described previously [20], a single immunisation with 3 × 30 μL of the emulsion was performed subcutaneously into several sites at the base of the tail of SJL/J mice. Immediately after immunisation (day 0) and 48 h thereafter, 400 ng of pertussis toxin (US Biological, Swampscott, MA, USA) was injected intraperitoneally. Only mice that developed EAE symptoms were randomised into the experimental groups. Mice were examined every other day for signs of EAE, and the severity of disease was graded using the following scale: 0, normal; 1, limp tail; 2, wobbly gait; 3, hind limb weakness; 4, hind limb paralysis; 5, tetraparalysis/death. In order to examine immunological parameters, there were four experimental groups with 20 mice per group: control, EAE, EAE + thymulin, and EAE + PBCA–thymulin. Animals were killed in subgroups of five on days 12, 18, and 25. Thymulin or nanoparticle-bound thymulin were administered intraperitoneally at a dose of 1.5 mg/kg (in the case of PBCA–thymulin—an equivalent of the quantity of pure thymulin), every other day during the whole observation period (25 days), and the first injection was in the day of immunisation (day 0).

### 4.4. Nanoparticle Size and Morphology

The size and morphology of the nanoparticles were examined by transmission electron microscopy (TEM) (JEM-100B; Jeol, Japan) with uranyl acetate and lead citrate contrast staining.

### 4.5. Blood Plasma

Blood was collected following decapitation of the animals. Blood samples were kept for 3–5 h at 4 °C and centrifuged at 200× *g* to determine plasma cytokines by ELISA assay.

### 4.6. Spleen Cells

Spleen cells were isolated from the spleen in medium 199 (Sigma-Aldrich, St. Louis, MO, USA) containing 1% 1 M HEPES solution, 100 µg/mL streptomycin, and 10% foetal bovine serum. Erythrocytes were lysed in Tris-buffered ammonium chloride (0.01 M Tris-HCl with 0.15 M NaCl and 0.83% NH_4_Cl at a ratio of 9:1). After washing, the samples were stored at a concentration of 1 × 10^8^ cells/mL in RPMI 1640 medium at −20 °C until a Western blot assay.

### 4.7. ELISA

ELISA was used to determine plasma cytokine concentrations. ELISA Development Kits for mouse TNF-α, IL-6, IL-10, IL-17, and IFN-γ (PeproTech, Rocky Hill, NJ, USA) were used. The following solution was applied to visualize binding: 100 μL of 2,2-azinobis(3-ethylbenzthiazoline-6-sulphonate) (ABTS) green dye (Sigma, USA) dissolved in 0.05 M citrate buffer (pH 4.0) with 0.01% H2O2. The optical density was measured at 405 nm with a plate spectrophotometer (Multiscan EX, Thermo Electron Corporation, Vintaa, Finland). The sensitivities for TNFα, IL-6, IL-17A, IL-10, and IFNγ kits were 32, 32, 32, 32, and 16 pg/mL, respectively. Measurements were made at different times after immunization (day 0). The values are expressed as an average of the means from five independent experiments (ng/mL) ± SEM; within each separate measurement, the samples were concurrently measured in quadruplicate. The averaged values from five experiments were analysed to determine the significance of the differences between groups (*n* = 5).

### 4.8. Western Blot Analysis

To prepare specimens, 1 × 10^8^ splenic cells were lysed using Triton^®^ X-100 lysis buffer, containing 50 mM Tris-HCl (pH 7.4), 150 mM NaCl, 1% Triton X-100, and 5 mM EDTA (Thermo Fisher GmbH, Kandel, Germany). The total protein concentration in each sample was then determined using the Bradford method. Subsequently, the proteins in each sample were precipitated with acetone, solubilised, boiled for 5 min, and stored at −70 °C. The proteins (10 μg of total protein per lane) were resolved by electrophoresis on a 10% polyacrylamide gel and transferred to a nitrocellulose membrane (Amersham/GE Healthcare, Chicago, IL, USA) in a Trans-Blot chamber (Bio-Rad, Berkeley, CA, USA). After blocking, the membrane was exposed to an antibody against one of the following mouse proteins for 2 h: heat shock protein (Hsp)70 (rabbit anti-Hsp72 antibody, clone SPA-812, inducible form; Enzo, Lausen, Switzerland; diluted 1:1000), phospho-NF-κB (rabbit anti-phospho-NF-κB p65 (Ser276) antibody; Cell Signaling Technology, Leiden, The Netherlands; diluted 1:1000), phospho-NF-κB (rabbit anti-phospho-NF-κB p65 (Ser536) antibody; Cell Signaling Technology, USA; diluted 1:1000), NF-κB (rabbit anti-NF-κB p65 antibody; Cell Signaling Technology; diluted 1:1000), phospho-IKKα/β (rabbit anti-phospho-IKKα/β antibody (Ser176/180); Cell Signaling Technology; diluted 1:1000), IKKβ (rabbit anti-IKKβ antibody; Cell Signaling Technology; diluted 1:1000), phospho-SAPK/JNK (rabbit anti-phospho-SAPK/JNK (Thr183/Tyr185) antibody; Cell Signaling Technology; diluted 1:1000), SAPK/JNK (rabbit anti-SAPK/JNK antibody; Cell Signaling Technology; diluted 1:1000), phospho-p53 (rabbit anti-phospho-p53 (Ser46) antibody; Cell Signaling Technology; diluted 1:1000), p53 (rabbit anti-p53 (1C12) antibody; Cell Signaling Technology; diluted 1:1000), or caspase-3 (rabbit anti-caspase-3 (8G10) antibody; Cell Signaling Technology; diluted 1:1000). After washing, the membrane was incubated for 1 h with a biotinylated goat anti-rabbit antibody (Biotin-SP (long spacer) AffiniPure Goat Anti-Rabbit IgG (H + L); Jackson ImmunoResearch, USA; diluted 1:200,000), before being exposed to 0.1 µg/mL peroxidase-conjugated streptavidin (Jackson ImmunoResearch, Cambridge, UK) for 1 h. To control for variations in protein loading, an antibody raised against a synthetic peptide corresponding to an amino acid sequence near the carboxyl-terminus of human glyceraldehyde-3-phosphate dehydrogenase (GAPDH) was used (rabbit anti-GAPDH (14C10) monoclonal antibody; Cell Signaling Technology; diluted 1:1000). ECL Plus chemiluminescence reagents (Amersham/GE Healthcare) and Hyperfilm ECL (Amersham/GE Healthcare) were then used to develop the blots according to the manufacturer’s instructions. The developed films were observed with a Vilber Lourmat (Marne-la-Vallée, France) TFX-35.WL transilluminator. Quantitative evaluation of protein bands was subsequently performed using QAPA software v. 3.7 (Institute of Cell Biophysics, Pushchino, Russia).

### 4.9. Statistical Analysis

Statistical analysis was performed using Statistica 6.0 software (StatSoft, Tulsa, OK, USA). For analysis of the severity score data, the Mann–Whitney U-test was used, because the distribution was not normal. *p*-values ≤ 0.05 were considered significant. In other cases Student’s *t*-tests were used to determine the significance of differences among the groups, with *p*-values ≤ 0.05 considered significant. All values are expressed as the mean ± standard error of the mean.

## 5. Conclusions

This is the first study to demonstrate that thymulin, free or bound to PBCA nanoparticles, may alleviate symptoms and dampen the peripheral autoimmune inflammatory response in chronic experimental autoimmune encephalomyelitis characterised by intermittent relapses and remissions. The increase in the blood content of thymulin, which is depleted with age, and the prolongation of its blood half-life, alone or in combination with other treatments, may be a prospective strategy for treatment of chronic inflammatory conditions.
